# Hexakis(1*H*-imidazole-κ*N* ^3^)nickel(II) bis­(2,4-dibromo-6-formyl­phenolate) *N*,*N*-dimethyl­formamide disolvate

**DOI:** 10.1107/S1600536808018989

**Published:** 2008-06-28

**Authors:** Yu Ding, Chunlian Li

**Affiliations:** a Department of Chemistry, Xiaogan University, Xiaogan, Hubei 432000, People’s Republic of China

## Abstract

In the cation of the title compound, [Ni(C_3_H_4_N_2_)_6_](C_7_H_3_Br_2_O_2_)_2_·2C_3_H_7_NO, the Ni^II^ ion lies on an inversion center and is coordinated in a slightly distorted octa­hedral environment by six N atoms from six imidazole ligands. In the crystal structure, cations, anions and solvent mol­ecules are linked by inter­molecular N—H⋯O hydrogen bonds into one-dimensional chains along [010]. In addition, the crystal structure is stabilized by weak C—H⋯O and C—H⋯N hydrogen bonds.

## Related literature

For related literature, see: Gelman *et al.* (2002 ▶).
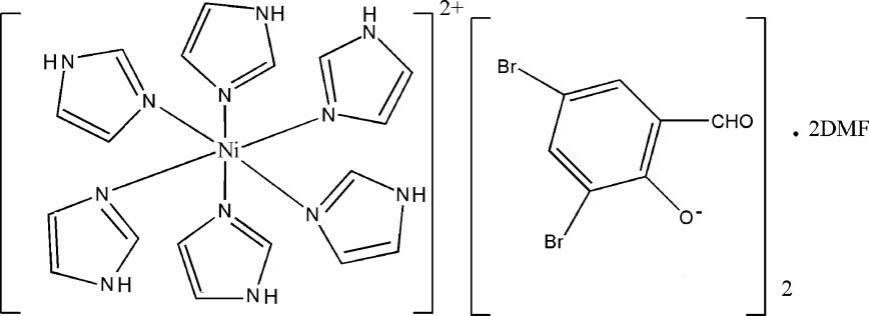


## Experimental

### 

#### Crystal data

[Ni(C_3_H_4_N_2_)_6_](C_7_H_3_Br_2_O_2_)_2_·2C_3_H_7_NO
*M* *_r_* = 1171.22
Monoclinic, 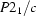

*a* = 14.7271 (13) Å
*b* = 9.0221 (8) Å
*c* = 18.1143 (16) Å
β = 100.408 (2)°
*V* = 2367.2 (4) Å^3^
*Z* = 2
Mo *K*α radiation
μ = 3.84 mm^−1^
*T* = 292 (2) K
0.25 × 0.20 × 0.20 mm

#### Data collection

Bruker SMART CCD diffractometer
Absorption correction: multi-scan (*SADABS*; Bruker, 2001 ▶) *T*_min_ = 0.308, *T*_max_ = 0.392 (expected range = 0.365–0.464)
13477 measured reflections
5147 independent reflections
3646 reflections with *I* > 2σ(*I*)
*R*_int_ = 0.031

#### Refinement

*R*[*F* ^2^ > 2σ(*F* ^2^)] = 0.043
*wR*(*F* ^2^) = 0.113
*S* = 1.01
5147 reflections
288 parameters
H-atom parameters constrained
Δρ_max_ = 0.57 e Å^−3^
Δρ_min_ = −0.32 e Å^−3^

### 

Data collection: *SMART* (Bruker, 2001 ▶); cell refinement: *SAINT* (Bruker, 2001 ▶); data reduction: *SAINT*; program(s) used to solve structure: *SHELXS97* (Sheldrick, 2008 ▶); program(s) used to refine structure: *SHELXL97* (Sheldrick, 2008 ▶); molecular graphics: *SHELXTL* (Sheldrick, 2008 ▶) and *PLATON* (Spek, 2003 ▶); software used to prepare material for publication: *SHELXTL*.

## Supplementary Material

Crystal structure: contains datablocks I, global. DOI: 10.1107/S1600536808018989/lh2618sup1.cif

Structure factors: contains datablocks I. DOI: 10.1107/S1600536808018989/lh2618Isup2.hkl

Additional supplementary materials:  crystallographic information; 3D view; checkCIF report

## Figures and Tables

**Table:** 

Ni1—N5	2.121 (2)
Ni1—N3	2.128 (2)
Ni1—N1	2.138 (2)

**Table:** 

N5—Ni1—N5^i^	180
N5—Ni1—N3^i^	91.41 (9)
N5—Ni1—N3	88.59 (9)
N3^i^—Ni1—N3	180
N5—Ni1—N1^i^	89.86 (9)
N3—Ni1—N1^i^	91.48 (9)
N5—Ni1—N1	90.14 (9)
N3—Ni1—N1	88.52 (9)
N1^i^—Ni1—N1	180

Symmetry code: (i) 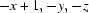.

**Table 2:** Hydrogen-bond geometry (Å, °)

*D*—H⋯*A*	*D*—H	H⋯*A*	*D*⋯*A*	*D*—H⋯*A*
N2—H2*A*⋯O3^ii^	0.86	1.92	2.764 (5)	169
N4—H4*A*⋯O2^iii^	0.86	1.85	2.703 (3)	170
N6—H6*A*⋯O2^iv^	0.86	1.97	2.772 (3)	155
C7—H7⋯N1^i^	0.93	2.57	3.076 (4)	115
C8—H8⋯O1^v^	0.93	2.59	3.264 (5)	130
C3—H3⋯N3	0.93	2.57	3.053 (4)	113

Symmetry codes: (i) 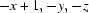; (ii) 
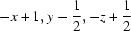
; (iii) 
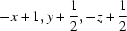
; (iv) 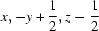; (v) 
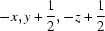
.

**Fig. 1.**
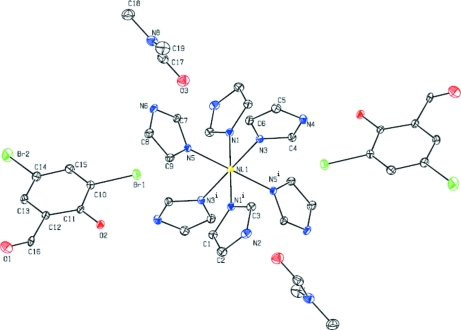
The molecular structure of (I), showing displacement ellipsoids at the 30% probability level [symmetry code: (i) -x+1, -y, -z]. H atoms have been omitted.

**Fig. 2.**
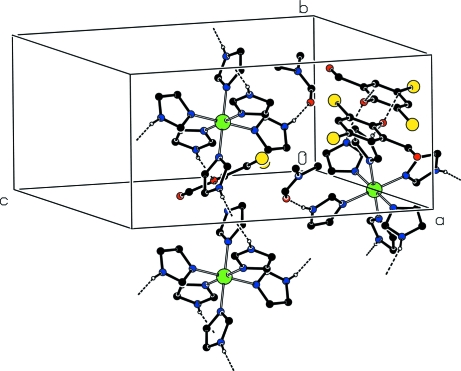
Part of the crystal structure showing hydrogen bonds as dashed lines.

## supplementary crystallographic information

### Comment

Due to the weak coordination strength of dibromosalicylaldehydenate anions with transition metals, the dibromosalicylaldehydenate usually acts as the counterbalance of the charge. Herein, we report the crystal structure of such a compound, [Ni(Im)_6_](DBSH)_2_ 2DMF, (I), (Im = imidazole; H_2_DBSH =3,5-dibromosalicylaldehyde; DMF =*N*,*N*-dimethylformamide). The molecular structure of (I) is shown in Fig.1. The Ni^II^ ion lying on an inversion center has a distorted octahedral geometry being coordinated by six N atoms from six imidazole ligands. Atoms N3, N3^i^, N5 & N5^i^comprise the equatorial plane, whereas the other two N atoms (N1 & N1^i^) occupy the axial positions (symmetry code as is Table 1). The Ni—N distances (Table 1), and the average Ni—N bond length of 2.12 Å, are longer than the Ni—N distances in [Ni(nap)(bip)](Cl)(nap = 1-naphthyl; bip =2,2^'^-bipyridine-N,*N*^'^; Ni—N 1.919 (8) Å) (Gelman *et al.*, 2002). As shown in Fig.2, an organic cation layer is linked to an inorganic anionic layer through a series of N—H···O, C—H···O and C—H···N hydrogen bonds (Table 2), and adjacent 3,5-dibromosalicylaldehydenate anions are antiparallel. The hydrogen bonds stabilize the crystal structure.

### Experimental

The title compound was prepared by adding Ni(Ac)_2_^.^2H_2_O (0.110 g, 0.5 mmol) to a solution of H_2_(DBSH) 0.122 mg (0.5 mmol) in methanol (20 mL) and DMF (20 ml). After stirring the mixture for 2 h, the solution was filtered and kept for several days at ambient temperature to evaporate. Brown block-like crystals were obtained.

### Refinement

All H atoms were placed in geometrically idealized positions and refined in the riding- model approximation, with N-H = 0.86 Å and C–H = 0.93 or 0.96 Å and *U*_iso_(H) = 1.2U_eq_(C,N) or 1.5U_eq_(C_methyl_)

### Crystal data

**Table:**

[Ni(C_3_H_4_N_2_)_6_](C_7_H_3_Br_2_O_2_)_2_·2C_3_H_7_NO	*F*_000_ = 1172
*M**_r_* = 1171.22	*D*_x_ = 1.643 Mg m^−^^3^
Monoclinic, *P*2_1_/*c*	Mo *K*α radiation λ = 0.71073 Å
Hall symbol: -P 2ybc	Cell parameters from 3743 reflections
*a* = 14.7271 (13) Å	θ = 2.3–25.2º
*b* = 9.0221 (8) Å	µ = 3.84 mm^−^^1^
*c* = 18.1143 (16) Å	*T* = 292 (2) K
β = 100.408 (2)º	Block, brown
*V* = 2367.2 (4) Å^3^	0.25 × 0.20 × 0.20 mm
*Z* = 2	

### Data collection

**Table:**

Bruker SMART CCD diffractometer	5147 independent reflections
Radiation source: fine-focus sealed tube	3646 reflections with *I* > 2σ(*I*)
Monochromator: graphite	*R*_int_ = 0.031
*T* = 292(2) K	θ_max_ = 27.0º
φ and ω scans	θ_min_ = 1.4º
Absorption correction: multi-scan(SADABS; Bruker, 2001)	*h* = −18→10
*T*_min_ = 0.309, *T*_max_ = 0.392	*k* = −11→10
13477 measured reflections	*l* = −23→23

### Refinement

**Table:**

Refinement on *F*^2^	Secondary atom site location: difference Fourier map
Least-squares matrix: full	Hydrogen site location: inferred from neighbouring sites
*R*[*F*^2^ > 2σ(*F*^2^)] = 0.043	H-atom parameters constrained
*wR*(*F*^2^) = 0.113	*w* = 1/[σ^2^(*F*_o_^2^) + (0.0625*P*)^2^] where *P* = (*F*_o_^2^ + 2*F*_c_^2^)/3
*S* = 1.01	(Δ/σ)_max_ = 0.001
5147 reflections	Δρ_max_ = 0.57 e Å^−^^3^
288 parameters	Δρ_min_ = −0.32 e Å^−^^3^
Primary atom site location: structure-invariant direct methods	Extinction correction: none

### Special details

**Table:**

Geometry. All e.s.d.'s (except the e.s.d. in the dihedral angle between two l.s. planes) are estimated using the full covariance matrix. The cell e.s.d.'s are taken into account individually in the estimation of e.s.d.'s in distances, angles and torsion angles; correlations between e.s.d.'s in cell parameters are only used when they are defined by crystal symmetry. An approximate (isotropic) treatment of cell e.s.d.'s is used for estimating e.s.d.'s involving l.s. planes.
Refinement. Refinement of *F*^2^ against ALL reflections. The weighted *R*-factor *wR* and goodness of fit *S* are based on *F*^2^, conventional *R*-factors *R* are based on *F*, with *F* set to zero for negative *F*^2^. The threshold expression of *F*^2^ > σ(*F*^2^) is used only for calculating *R*-factors(gt) *etc*. and is not relevant to the choice of reflections for refinement. *R*-factors based on *F*^2^ are statistically about twice as large as those based on *F*, and *R*- factors based on ALL data will be even larger.

### Fractional atomic coordinates and isotropic or equivalent isotropic displacement parameters (Å^2^)

**Table:**

	*x*	*y*	*z*	*U*_iso_*/*U*_eq_	
Ni1	0.5000	0.0000	0.0000	0.03274 (14)	
Br1	0.24272 (2)	0.17200 (5)	0.27116 (2)	0.06303 (15)	
Br2	−0.11882 (4)	0.03677 (8)	0.12533 (3)	0.1107 (2)	
N1	0.52342 (17)	−0.0719 (3)	0.11435 (13)	0.0402 (6)	
N2	0.5794 (2)	−0.0782 (4)	0.23540 (15)	0.0586 (8)	
H2A	0.6097	−0.0524	0.2786	0.070*	
N3	0.56038 (16)	0.2073 (3)	0.03727 (13)	0.0393 (6)	
N4	0.6664 (2)	0.3744 (3)	0.07883 (15)	0.0524 (7)	
H4A	0.7192	0.4175	0.0890	0.063*	
N5	0.36975 (16)	0.0871 (3)	0.01215 (13)	0.0379 (6)	
N6	0.25325 (17)	0.2412 (3)	−0.00608 (16)	0.0487 (7)	
H6A	0.2142	0.3053	−0.0279	0.058*	
C16	−0.0057 (2)	−0.0903 (4)	0.41722 (18)	0.0451 (8)	
H16	0.0403	−0.0923	0.4598	0.054*	
N8	0.2044 (3)	0.7094 (4)	0.1345 (2)	0.0744 (9)	
O1	−0.0693 (3)	−0.1323 (4)	0.42179 (16)	0.0882 (9)	
O2	0.17355 (14)	0.0277 (2)	0.40408 (11)	0.0457 (5)	
O3	0.3141 (2)	0.5358 (4)	0.13585 (18)	0.0927 (10)	
C1	0.4906 (2)	−0.1939 (4)	0.1457 (2)	0.0545 (9)	
H1	0.4506	−0.2635	0.1195	0.065*	
C2	0.5247 (3)	−0.1991 (5)	0.2203 (2)	0.0647 (10)	
H2	0.5130	−0.2710	0.2542	0.078*	
C3	0.5773 (2)	−0.0056 (4)	0.17004 (18)	0.0498 (8)	
H3	0.6100	0.0809	0.1651	0.060*	
C4	0.6477 (2)	0.2437 (4)	0.04387 (17)	0.0465 (8)	
H4	0.6916	0.1858	0.0264	0.056*	
C5	0.5854 (3)	0.4248 (4)	0.0949 (2)	0.0609 (10)	
H5	0.5761	0.5129	0.1192	0.073*	
C6	0.5208 (2)	0.3220 (4)	0.06879 (19)	0.0517 (8)	
H6	0.4586	0.3285	0.0719	0.062*	
C7	0.3228 (2)	0.1833 (3)	−0.03379 (18)	0.0434 (7)	
H7	0.3367	0.2082	−0.0803	0.052*	
C8	0.2548 (2)	0.1816 (4)	0.0622 (2)	0.0590 (9)	
H8	0.2149	0.2021	0.0952	0.071*	
C9	0.3262 (2)	0.0860 (4)	0.07290 (19)	0.0522 (8)	
H9	0.3435	0.0277	0.1154	0.063*	
C10	0.1241 (2)	0.0919 (4)	0.27542 (18)	0.0461 (7)	
C11	0.1103 (2)	0.0322 (3)	0.34538 (18)	0.0400 (7)	
C12	0.0195 (2)	−0.0232 (3)	0.34397 (18)	0.0455 (8)	
C13	−0.0480 (2)	−0.0201 (4)	0.2791 (2)	0.0546 (9)	
H13	−0.1067	−0.0573	0.2801	0.066*	
C14	−0.0282 (3)	0.0373 (4)	0.2142 (2)	0.0584 (9)	
C15	0.0579 (2)	0.0958 (4)	0.21212 (18)	0.0565 (9)	
H15	0.0707	0.1374	0.1681	0.068*	
C19	0.2451 (3)	0.6031 (5)	0.1056 (2)	0.0727 (11)	
H19	0.2195	0.5749	0.0569	0.087*	
C17	0.2366 (4)	0.7550 (7)	0.2115 (3)	0.1150 (19)	
H17A	0.1915	0.7283	0.2414	0.173*	
H17B	0.2455	0.8604	0.2134	0.173*	
H17C	0.2940	0.7065	0.2308	0.173*	
C18	0.1235 (4)	0.7834 (6)	0.0939 (3)	0.1070 (17)	
H18A	0.1126	0.7516	0.0425	0.161*	
H18B	0.1332	0.8886	0.0961	0.161*	
H18C	0.0710	0.7590	0.1162	0.161*	

### Atomic displacement parameters (Å^2^)

**Table:**

	*U*^11^	*U*^22^	*U*^33^	*U*^12^	*U*^13^	*U*^23^
Ni1	0.0284 (3)	0.0338 (3)	0.0357 (3)	0.0004 (2)	0.0047 (2)	−0.0001 (2)
Br1	0.0482 (2)	0.0825 (3)	0.0575 (2)	−0.01576 (18)	0.00701 (17)	0.00634 (18)
Br2	0.0836 (4)	0.1536 (5)	0.0743 (3)	−0.0336 (3)	−0.0404 (3)	0.0199 (3)
N1	0.0382 (14)	0.0433 (15)	0.0396 (14)	0.0043 (12)	0.0087 (11)	0.0020 (12)
N2	0.067 (2)	0.071 (2)	0.0378 (15)	0.0125 (17)	0.0083 (14)	0.0040 (15)
N3	0.0374 (14)	0.0380 (14)	0.0418 (14)	−0.0036 (11)	0.0047 (11)	−0.0010 (11)
N4	0.0472 (17)	0.0496 (17)	0.0562 (17)	−0.0136 (13)	−0.0017 (13)	−0.0045 (14)
N5	0.0311 (13)	0.0386 (14)	0.0442 (14)	0.0012 (10)	0.0070 (11)	−0.0019 (11)
N6	0.0337 (14)	0.0465 (16)	0.0626 (17)	0.0100 (12)	0.0001 (12)	−0.0058 (14)
C16	0.0262 (15)	0.059 (2)	0.0451 (18)	−0.0011 (15)	−0.0081 (13)	−0.0160 (16)
N8	0.088 (3)	0.061 (2)	0.069 (2)	−0.0127 (19)	0.0011 (19)	−0.0037 (18)
O1	0.111 (3)	0.091 (2)	0.0664 (18)	−0.015 (2)	0.0265 (18)	0.0002 (16)
O2	0.0361 (12)	0.0528 (14)	0.0436 (12)	0.0005 (9)	−0.0051 (10)	−0.0011 (10)
O3	0.099 (3)	0.093 (2)	0.079 (2)	0.002 (2)	−0.0024 (19)	0.0033 (18)
C1	0.050 (2)	0.056 (2)	0.058 (2)	0.0017 (16)	0.0107 (16)	0.0111 (17)
C2	0.070 (3)	0.075 (3)	0.053 (2)	0.004 (2)	0.0205 (19)	0.019 (2)
C3	0.054 (2)	0.055 (2)	0.0405 (17)	0.0034 (16)	0.0089 (15)	−0.0004 (16)
C4	0.0444 (19)	0.047 (2)	0.0474 (18)	−0.0039 (15)	0.0072 (14)	−0.0012 (16)
C5	0.061 (2)	0.042 (2)	0.077 (3)	−0.0013 (17)	0.005 (2)	−0.0146 (19)
C6	0.0444 (19)	0.0440 (19)	0.066 (2)	0.0017 (15)	0.0070 (16)	−0.0112 (16)
C7	0.0353 (16)	0.0476 (19)	0.0443 (17)	0.0019 (14)	−0.0006 (13)	−0.0022 (15)
C8	0.050 (2)	0.067 (2)	0.066 (2)	0.0175 (18)	0.0270 (18)	0.0084 (19)
C9	0.050 (2)	0.055 (2)	0.055 (2)	0.0130 (16)	0.0214 (16)	0.0123 (16)
C10	0.0384 (17)	0.0481 (19)	0.0507 (18)	−0.0033 (14)	0.0051 (14)	0.0018 (15)
C11	0.0319 (16)	0.0354 (17)	0.0498 (18)	0.0041 (12)	−0.0002 (13)	−0.0057 (13)
C12	0.0368 (17)	0.048 (2)	0.0504 (19)	0.0013 (14)	0.0029 (14)	−0.0005 (15)
C13	0.0382 (19)	0.057 (2)	0.064 (2)	−0.0085 (15)	−0.0036 (16)	−0.0035 (17)
C14	0.052 (2)	0.065 (2)	0.050 (2)	−0.0074 (17)	−0.0156 (16)	0.0011 (17)
C15	0.059 (2)	0.061 (2)	0.0443 (18)	−0.0076 (18)	−0.0037 (16)	0.0047 (17)
C19	0.088 (3)	0.068 (3)	0.058 (2)	−0.020 (2)	0.004 (2)	0.002 (2)
C17	0.147 (5)	0.104 (4)	0.090 (4)	−0.009 (4)	0.010 (3)	−0.022 (3)
C18	0.108 (4)	0.073 (3)	0.126 (4)	0.000 (3)	−0.015 (3)	0.004 (3)

### Geometric parameters (Å, °)

**Table:**

Ni1—N5	2.121 (2)	O2—C11	1.282 (4)
Ni1—N5^i^	2.121 (2)	O3—C19	1.224 (5)
Ni1—N3^i^	2.128 (2)	C1—C2	1.355 (5)
Ni1—N3	2.128 (2)	C1—H1	0.9300
Ni1—N1^i^	2.138 (2)	C2—H2	0.9300
Ni1—N1	2.138 (2)	C3—H3	0.9300
Br1—C10	1.905 (3)	C4—H4	0.9300
Br1—H8	3.1509	C5—C6	1.351 (5)
Br2—C14	1.896 (3)	C5—H5	0.9300
N1—C3	1.309 (4)	C6—H6	0.9300
N1—C1	1.366 (4)	C7—H7	0.9300
N2—C3	1.348 (4)	C8—C9	1.346 (5)
N2—C2	1.354 (5)	C8—H8	0.9300
N2—H2A	0.8600	C9—H9	0.9300
N3—C4	1.312 (4)	C10—C15	1.365 (4)
N3—C6	1.362 (4)	C10—C11	1.425 (4)
N4—C4	1.343 (4)	C11—C12	1.424 (4)
N4—C5	1.357 (4)	C12—C13	1.396 (5)
N4—H4A	0.8600	C13—C14	1.364 (5)
N5—C7	1.310 (4)	C13—H13	0.9300
N5—C9	1.370 (4)	C14—C15	1.381 (5)
N6—C7	1.327 (4)	C15—H15	0.9300
N6—C8	1.345 (4)	C19—H19	0.9300
N6—H6A	0.8600	C17—H17A	0.9600
C16—O1	1.028 (4)	C17—H17B	0.9600
C16—C12	1.563 (5)	C17—H17C	0.9600
C16—H16	0.9300	C18—H18A	0.9600
N8—C19	1.290 (6)	C18—H18B	0.9600
N8—C18	1.446 (6)	C18—H18C	0.9600
N8—C17	1.450 (6)		
			
N5—Ni1—N5^i^	180	N3—C4—H4	124.0
N5—Ni1—N3^i^	91.41 (9)	N4—C4—H4	124.0
N5^i^—Ni1—N3^i^	88.59 (9)	C6—C5—N4	106.5 (3)
N5—Ni1—N3	88.59 (9)	C6—C5—H5	126.7
N5^i^—Ni1—N3	91.41 (9)	N4—C5—H5	126.7
N3^i^—Ni1—N3	180	C5—C6—N3	110.0 (3)
N5—Ni1—N1^i^	89.86 (9)	C5—C6—H6	125.0
N5^i^—Ni1—N1^i^	90.14 (9)	N3—C6—H6	125.0
N3^i^—Ni1—N1^i^	88.52 (9)	N5—C7—N6	112.0 (3)
N3—Ni1—N1^i^	91.48 (9)	N5—C7—H7	124.0
N5—Ni1—N1	90.14 (9)	N6—C7—H7	124.0
N5^i^—Ni1—N1	89.86 (9)	N6—C8—C9	105.8 (3)
N3^i^—Ni1—N1	91.48 (9)	N6—C8—H8	127.1
N3—Ni1—N1	88.52 (9)	C9—C8—H8	127.1
N1^i^—Ni1—N1	180	C8—C9—N5	110.3 (3)
C10—Br1—H8	96.9	C8—C9—H9	124.9
C3—N1—C1	105.0 (3)	N5—C9—H9	124.9
C3—N1—Ni1	125.3 (2)	C15—C10—C11	124.2 (3)
C1—N1—Ni1	129.8 (2)	C15—C10—Br1	118.5 (3)
C3—N2—C2	107.2 (3)	C11—C10—Br1	117.3 (2)
C3—N2—H2A	126.4	O2—C11—C12	122.9 (3)
C2—N2—H2A	126.4	O2—C11—C10	123.4 (3)
C4—N3—C6	105.0 (3)	C12—C11—C10	113.7 (3)
C4—N3—Ni1	126.5 (2)	C13—C12—C11	122.1 (3)
C6—N3—Ni1	127.9 (2)	C13—C12—C16	118.7 (3)
C4—N4—C5	106.5 (3)	C11—C12—C16	119.2 (3)
C4—N4—H4A	126.8	C14—C13—C12	120.2 (3)
C5—N4—H4A	126.8	C14—C13—H13	119.9
C7—N5—C9	104.2 (3)	C12—C13—H13	119.9
C7—N5—Ni1	124.2 (2)	C13—C14—C15	120.6 (3)
C9—N5—Ni1	130.1 (2)	C13—C14—Br2	120.2 (3)
C7—N6—C8	107.7 (3)	C15—C14—Br2	119.1 (3)
C7—N6—H6A	126.1	C10—C15—C14	119.2 (3)
C8—N6—H6A	126.1	C10—C15—H15	120.4
O1—C16—C12	124.8 (3)	C14—C15—H15	120.4
O1—C16—H16	117.6	O3—C19—N8	126.5 (4)
C12—C16—H16	117.6	O3—C19—H19	116.7
C19—N8—C18	122.5 (4)	N8—C19—H19	116.7
C19—N8—C17	120.4 (4)	N8—C17—H17A	109.5
C18—N8—C17	117.0 (4)	N8—C17—H17B	109.5
C2—C1—N1	110.3 (3)	H17A—C17—H17B	109.5
C2—C1—H1	124.8	N8—C17—H17C	109.5
N1—C1—H1	124.8	H17A—C17—H17C	109.5
N2—C2—C1	105.9 (3)	H17B—C17—H17C	109.5
N2—C2—H2	127.1	N8—C18—H18A	109.5
C1—C2—H2	127.1	N8—C18—H18B	109.5
N1—C3—N2	111.7 (3)	H18A—C18—H18B	109.5
N1—C3—H3	124.2	N8—C18—H18C	109.5
N2—C3—H3	124.2	H18A—C18—H18C	109.5
N3—C4—N4	112.1 (3)	H18B—C18—H18C	109.5
			
N5—Ni1—N1—C3	−102.9 (3)	C4—N4—C5—C6	−0.1 (4)
N5^i^—Ni1—N1—C3	77.1 (3)	N4—C5—C6—N3	0.6 (4)
N3^i^—Ni1—N1—C3	165.7 (3)	C4—N3—C6—C5	−0.9 (4)
N3—Ni1—N1—C3	−14.3 (3)	Ni1—N3—C6—C5	170.9 (2)
N5—Ni1—N1—C1	79.5 (3)	C9—N5—C7—N6	−0.4 (3)
N5^i^—Ni1—N1—C1	−100.5 (3)	Ni1—N5—C7—N6	−167.72 (19)
N3^i^—Ni1—N1—C1	−11.9 (3)	C8—N6—C7—N5	0.8 (4)
N3—Ni1—N1—C1	168.1 (3)	C7—N6—C8—C9	−0.9 (4)
N5—Ni1—N3—C4	−178.5 (3)	N6—C8—C9—N5	0.7 (4)
N5^i^—Ni1—N3—C4	1.5 (3)	C7—N5—C9—C8	−0.2 (4)
N1^i^—Ni1—N3—C4	−88.6 (3)	Ni1—N5—C9—C8	166.1 (2)
N1—Ni1—N3—C4	91.4 (3)	H8—Br1—C10—C15	−12.2
N5—Ni1—N3—C6	11.4 (3)	H8—Br1—C10—C11	168.2
N5^i^—Ni1—N3—C6	−168.6 (3)	C15—C10—C11—O2	179.1 (3)
N1^i^—Ni1—N3—C6	101.2 (3)	Br1—C10—C11—O2	−1.3 (4)
N1—Ni1—N3—C6	−78.8 (3)	C15—C10—C11—C12	−0.5 (5)
N3^i^—Ni1—N5—C7	−105.8 (2)	Br1—C10—C11—C12	179.1 (2)
N3—Ni1—N5—C7	74.2 (2)	O2—C11—C12—C13	−178.7 (3)
N1^i^—Ni1—N5—C7	−17.3 (2)	C10—C11—C12—C13	0.9 (4)
N1—Ni1—N5—C7	162.7 (2)	O2—C11—C12—C16	1.2 (4)
N3^i^—Ni1—N5—C9	90.3 (3)	C10—C11—C12—C16	−179.3 (3)
N3—Ni1—N5—C9	−89.7 (3)	O1—C16—C12—C13	−0.6 (6)
N1^i^—Ni1—N5—C9	178.8 (3)	O1—C16—C12—C11	179.6 (4)
N1—Ni1—N5—C9	−1.2 (3)	C11—C12—C13—C14	0.0 (5)
C3—N1—C1—C2	0.5 (4)	C16—C12—C13—C14	−179.8 (3)
Ni1—N1—C1—C2	178.4 (2)	C12—C13—C14—C15	−1.4 (6)
C3—N2—C2—C1	−0.6 (4)	C12—C13—C14—Br2	178.3 (3)
N1—C1—C2—N2	0.1 (4)	C11—C10—C15—C14	−0.8 (5)
C1—N1—C3—N2	−0.9 (4)	Br1—C10—C15—C14	179.6 (3)
Ni1—N1—C3—N2	−179.0 (2)	C13—C14—C15—C10	1.8 (6)
C2—N2—C3—N1	1.0 (4)	Br2—C14—C15—C10	−177.9 (3)
C6—N3—C4—N4	0.9 (4)	C18—N8—C19—O3	179.4 (5)
Ni1—N3—C4—N4	−171.08 (19)	C17—N8—C19—O3	−3.5 (7)
C5—N4—C4—N3	−0.5 (4)		

Symmetry codes: (i) −*x*+1, −*y*, −*z*.

### Hydrogen-bond geometry (Å, °)

**Table:**

*D*—H···*A*	*D*—H	H···*A*	*D*···*A*	*D*—H···*A*
N2—H2A···O3^ii^	0.86	1.92	2.764 (5)	169
N4—H4A···O2^iii^	0.86	1.85	2.703 (3)	170
N6—H6A···O2^iv^	0.86	1.97	2.772 (3)	155
C7—H7···N1^i^	0.93	2.57	3.076 (4)	115
C8—H8···O1^v^	0.93	2.59	3.264 (5)	130
C3—H3···N3	0.93	2.57	3.053 (4)	113

Symmetry codes: (ii) −*x*+1, *y*−1/2, −*z*+1/2; (iii) −*x*+1, *y*+1/2, −*z*+1/2; (iv) *x*, −*y*+1/2, *z*−1/2; (i) −*x*+1, −*y*, −*z*; (v) −*x*, *y*+1/2, −*z*+1/2.
